# Leptospirosis and its co-infections in India: prevalence, geographic distribution, and clinical implications

**DOI:** 10.1186/s12879-026-13642-7

**Published:** 2026-06-26

**Authors:** Gloris Joseph, Shrivathsa Kulavalli, Sabine Bélard, Kavitha Saravu

**Affiliations:** 1 Department of Infectious Diseases, Kasturba Medical College, Manipal Academy of Higher Education, Manipal, India; 2 Institute of Tropical Medicine, University of Tübingen, Tübingen, Germany; 3 German Center for Infection Research, DZIF Partner Site Tübingen, Tübingen, Germany

**Keywords:** Leptospirosis, Co-infections, Prevalence, Acute febrile illness (AFI)

## Abstract

**Background:**

Leptospirosis is a zoonotic disease prevalent in low and middle-income countries and tropical regions. It can be mimicked by endemic infectious diseases that present simultaneously, like dengue, malaria, melioidosis, or scrub typhus. However, there is a dearth of documented evidence about the frequency of coinfection, as well as related symptoms and mortality. The study aims to determine the frequency and geographical extent of coinfections in leptospirosis patients in India.

**Methodology:**

We systematically searched databases, including Scopus, PubMed, Embase, and Web of Science, for articles published between January 2010 and August 2024 assessing coinfections in leptospirosis patients in India. The eligible articles were evaluated for full text data extraction using a standardized methodology.

**Results:**

Of 804 articles screened 39 studies were included; overall these reported on 1565 leptospirosis cases with 236 (15.1%) cases with coinfections. Of these, seven observational studies assessed coinfections in 1318 leptospirosis-positive cases, identifying 113 (8.6%) coinfections: most commonly dengue (79; 69.9%), followed by hepatitis E (15; 13.2%), typhoid (9; 7.9%), and malaria (9; 7.9%). The most coinfection cases were reported from New Delhi (29.6%), followed by Kerala (21.6%), Karnataka (14.4%), Uttar Pradesh (12.2%), and Tamil Nadu (5.5%). Common symptoms included fever, vomiting, icterus, headache, thrombocytopenia, and transaminitis. Cases with coinfection had poorer outcomes with increased mortality compared to cases with isolated infection.

**Conclusion:**

In India, leptospirosis can present with co-infections such as dengue, hepatitis E, malaria, typhoid, and scrub typhus, complicating diagnosis due to overlapping clinical features. These coinfections are associated with unfavorable outcomes, including multi-organ dysfunction and increased mortality. Early diagnostic testing in febrile patients and timely, appropriate treatment are essential to improve patient outcomes.

**Trial registration:**

Clinical trial number: not applicable. Review protocol registration (Open Science Framework): DOI https://doi.org/10.17605/OSF.IO/AS9W5.

**Supplementary Information:**

The online version contains supplementary material available at https://doi.org/10.1186/s12879-026-13642-7.

## Introduction

Leptospirosis is a zoonotic disease caused by pathogenic *Leptospira spp.* transmitted through contact with the urine of infected animals (rodents, cats, pigs, dogs). The incidence of the disease, once thought rare, has been on the rise [1]. Monsoon flooding and heavy rainfall in coastal regions heighten the risk of leptospirosis exposure in the southern part of India [2]. The annual incidence of leptospirosis ranges from 0.1 to 1.0 per 100,000 in temperate zones and 10–100/100,000 in humid zones [3, 4]. An estimated 1 million cases resulting in nearly 60,000 deaths occur globally each year [5]. Coinfection is defined as the simultaneous infection of a host by multiple pathogen species [6]. The clinical features of leptospirosis are broad and often overlap with other infectious diseases, making it challenging to distinguish between mono- and co-infections. Undiagnosed coinfections, such as most commonly dengue, malaria, or scrub typhus, can complicate the further clinical course [7]. The more severe form of leptospirosis, icteric leptospirosis, also known as Weil’s syndrome, is characterized with jaundice and may progress to multi-organ failure, resulting in hepatic dysfunction with jaundice, renal impairment, and hemorrhagic manifestations. Mortality in severe leptospirosis patients ranges from 5% to 20% [8]. Mortality rates for severe leptospirosis associated with acute respiratory distress syndrome (ARDS) or severe pulmonary haemorrhage syndrome (SPHS) are even higher, with more than 50% [9]. Leptospirosis coinfections with dengue, scrub typhus, and malaria often result in misdiagnosis and increased disease severity [10]. Coinfections in leptospirosis patients hold significant clinical and public health importance due to their impact on diagnosis, disease severity, and patient outcome. However, knowledge on the frequency of leptospirosis coinfection with dengue, malaria, or scrub typhus and related patient outcomes is limited [5]. This review aimed at collating available published literature to determine the frequency and geographical extent of leptospirosis coinfections in India and outline related clinical features and disease outcomes.

## Methodology

This review was performed according to the PRISMA-ScR (Preferred Reporting Items for Systematic Reviews and Meta-Analyses Extension for Scoping Reviews) statement [11]. The following PICOS (Population (or Patient/Problem), Intervention (or Exposure), Comparison (or Control), Outcome(s), and Study design) format was followed: Population: leptospirosis patients with coinfection; Intervention/Exposure: not applicable; Comparison: leptospirosis mono-infection patients; Outcome: frequency of coinfections in leptospirosis patients, clinical symptomatology and mortality; Study design: observational studies, case reports, and case series. This review protocol was registered in the Open Science Framework (OSF) (Registration DOI: https://doi.org/10.17605/OSF.IO/AS9W5).

### Eligibility criteria & information sources

For this review, articles published in English between January 2010 and August 2024 reporting coinfections in leptospirosis cases, as well as acute febrile illness (AFI) studies documenting leptospirosis coinfections, regardless of diagnostic methods were considered, including original research articles, case series and/or case reports. The review articles, conference papers, participants from outside India, articles not addressing human disease, and those in other languages were excluded from the study. The literature search strategies developed were “((((India) AND (leptospirosis)) OR (leptospiral infection)) OR (Leptospira)) AND (co-infections)” based on PICOS using MeSH terms and all possible search commands from the four different databases: Scopus, PubMed, Embase, and Web of Science [Supplementary file 1 – Table S1].

### Selection of sources of evidence & data charting process

The studies were imported from databases in formats such as RIS or BibTeX. Based on the eligibility criteria, the identified and deduplicated articles were subjected to title and abstract screening using the Rayyan software. Full-text articles were screened, and data were extracted using a predefined data collection chart (Microsoft Excel) if the inclusion criteria were met. Full-text articles that were not freely accessible were requested twice directly from the corresponding authors, but they were excluded because of non-response. Literature search, screening, and data extraction were performed independently by two reviewers (GJ and SK), confirming all extracted data through cross-checking.

### Risk of bias assessment

The quality assessment was carried out independently by two reviewers (GJ and SK) using Joanna Briggs Institute’s (JBI) critical appraisal checklist for prevalence studies, case reports, and case series [12–14]. While performing risk of bias (RoB), each item was categorized into one of four choices: yes, no, unclear, or not applicable. Studies were rated as “low risk” (good) if they met ≥ 75% of the criteria, “moderate risk” (fair) if they met 50–74%, and “high risk” (poor) if they met < 50% [15, 16].

### Data extraction & synthesis of results

The following data were extracted: author, publication year, total number of patients, age and gender, location of the study, regional distribution of cases, study design, diagnostic tests, number of coinfections, clinical characteristics of the leptospirosis patients with coinfections, antibiotics used for the treatment, and fatalities of leptospirosis cases with coinfections. Extracted data was analysed in a descriptive way. No formal risk of bias assessment was conducted.

## Results

### Selection of sources of evidence

**Fig. 1:**
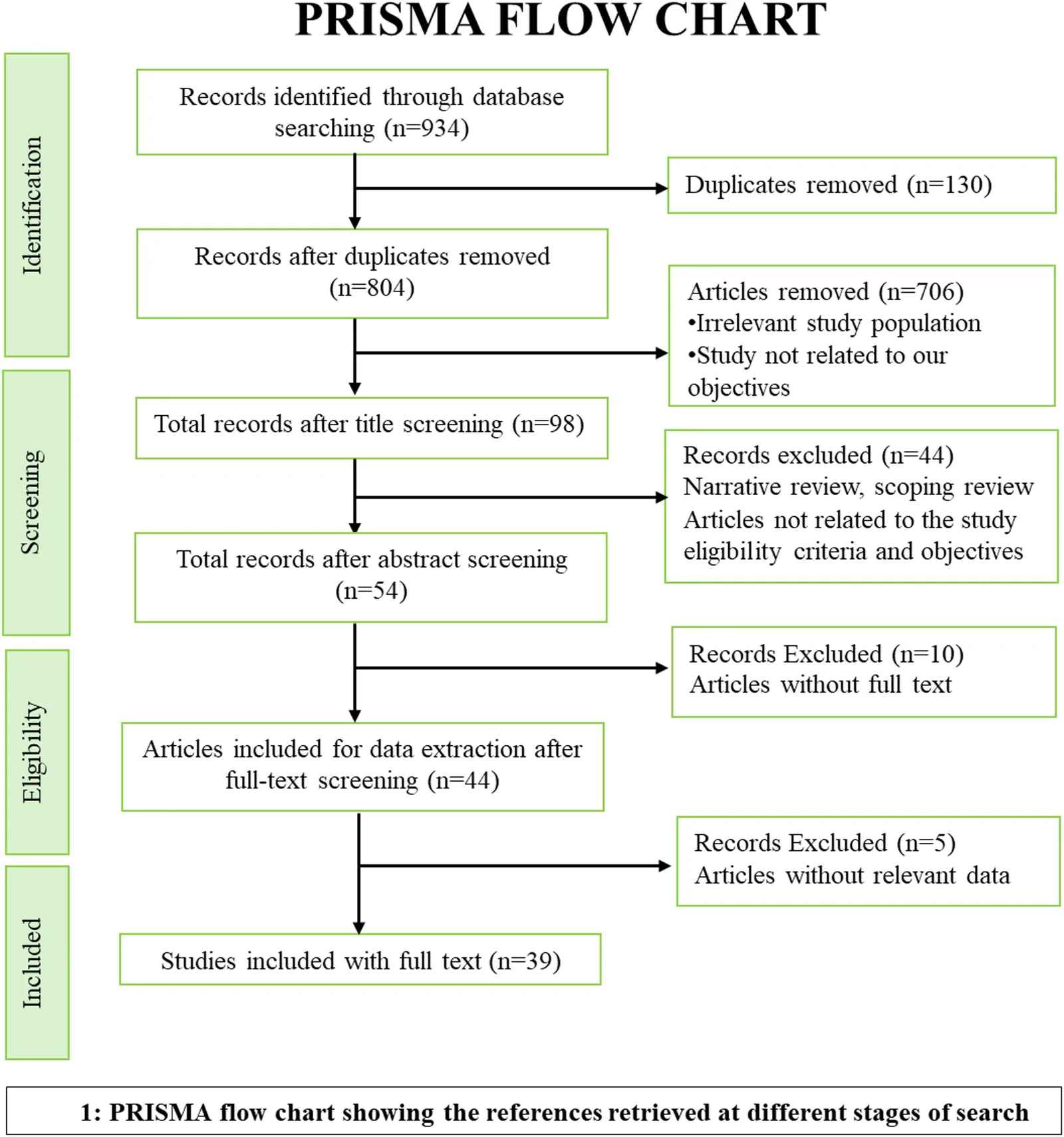
PRISMA flow chart showing the references retrieved at different stages of search

A total of 934 articles were identified from PubMed, Scopus, Embase, and Web of Science databases [Supplementary File 1 – Table S1]. After removing 130 duplicates, 804 articles were included for title and abstract screening. Thirty-nine articles reporting on leptospirosis patients with coinfections were primarily included in the review; however, 10 studies were excluded further on due to the unavailability of relevant data on coinfection with leptospirosis [PRISMA-ScR Fig. 1]. Among the 39 articles extracted from different databases, 20 were case reports, 15 were observational studies (of these, eight were studies on acute febrile illness (AFI), including leptospirosis cases with coinfections, and seven were observational studies of leptospirosis patients with coinfections), and four were case series.

### Synthesis of results

#### Diagnostic modalities

Various methods were used to diagnose leptospirosis. Among 1565 leptospirosis-positive cases across 39 studies, Enzyme Linked Immunosorbent Assay (ELISA) was the most common diagnostic test, identifying 1339 (85.5%) cases. The Microscopic Agglutination Test (MAT) identified 226 (14.4%) cases, PCR confirmed 56 (3.5%) cases, and dark-field microscopy identified a single case. The most frequently observed Leptospira serovars were icterohaemorrhagiae (13), followed by hebdomadis (5), pyrogenes (4), canicola (3), gripotyphosa (3), and patoc (1). The types and numbers of diagnostic assays used to detect coinfection varied substantially across studies, ranging from one assay to multiple molecular, serological, and culture-based methods, which may influence the detection ratio of coinfections. Diagnostic approaches for coinfections are summarized in Supplementary File 1 – Table S2. The Distribution of studies reporting leptospirosis coinfection cases confirmed by non-serological tests is depicted in Supplementary file 1 – Table S3. The total number of diagnostic assays per study ranged from 2 to 12 (mean ≈ 6.4), reflecting the broad differential diagnostic approach taken in suspected cases [Supplementary file 1 – Table S4]. The total number of pathogens or infections targeted for differential diagnosis in each study is depicted in Supplementary file 1 – Table S5.

#### Epidemiology and geographical distribution

**Fig. 2:**
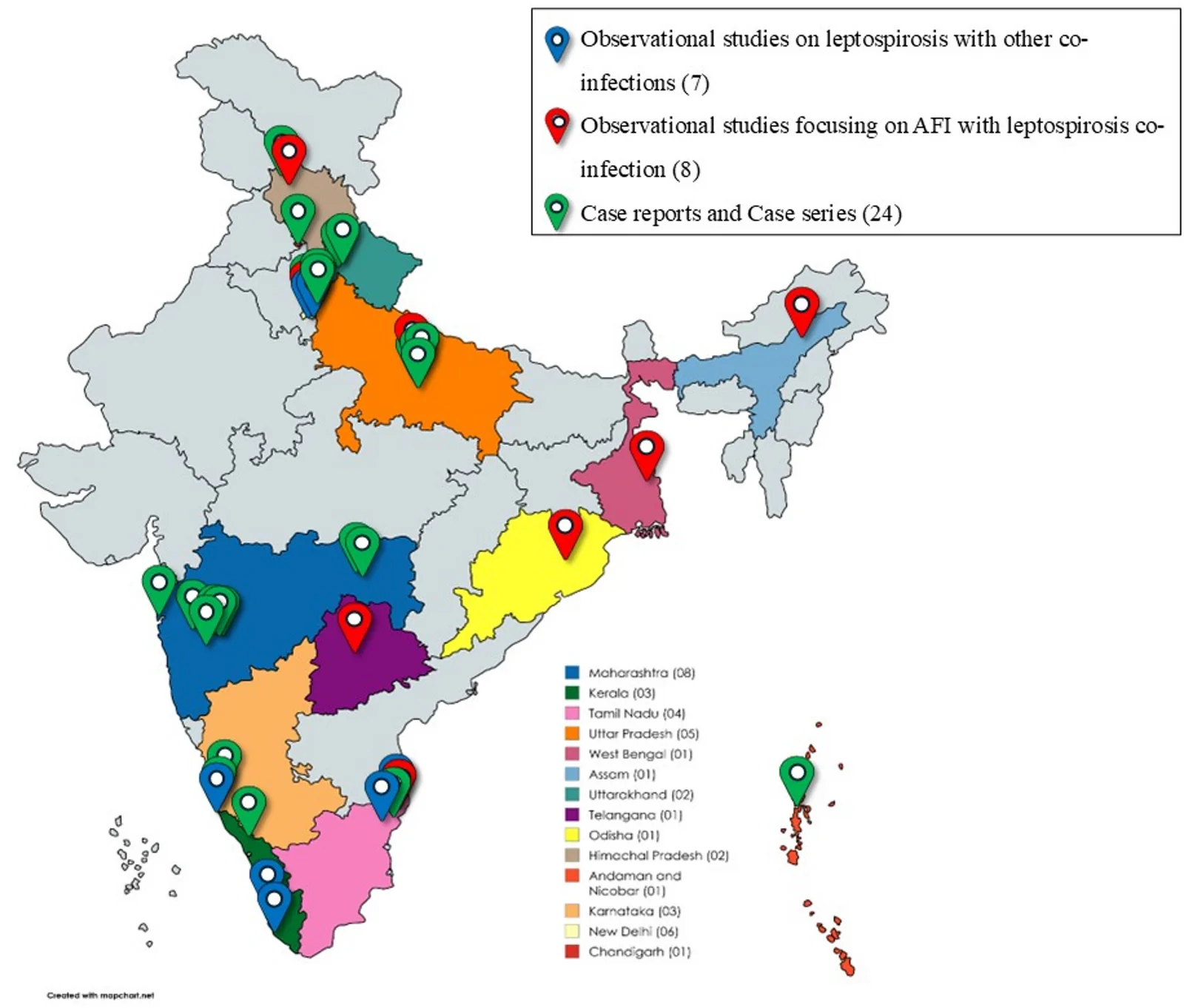
Geographic representations of publications of co-infection with leptospirosis across India. (The number of studies reported in each state is listed along with the respective states)

Analysis of leptospirosis coinfection cases across India revealed that New Delhi accounted for the highest proportion (29.6%) of leptospirosis patients with coinfection, followed by Kerala (21.6%), Karnataka (14.4%), Uttar Pradesh (12.2%), Tamil Nadu (5.5%), Maharashtra (4.2%), Assam (3.3%), Andaman & Nicobar (3.3%), Odisha (2.5%), Himachal Pradesh (1.6%), Telangana (1.2%), Uttarakhand (0.8%), and 0.4% each from Chandigarh and West Bengal. Figure 2 illustrates the geographical distribution of total co-infection cases from each study location. Most coinfected cases were reported in the monsoon season.

#### Co-infection and triple infection cases

The sample sizes across studies ranged from one to 12,783 individuals. Among these, 1565 cases were tested positive for leptospirosis, including 236 leptospirosis cases with coinfection. The mean age of the coinfected patients was 32 years; 62 were males, and 49 were females. The gender distribution was described in 29 articles, including 111 leptospirosis cases with co-infection. Excluding case series and reports, the seven observational studies on leptospirosis patients accounted for 1318 leptospirosis cases, with a total of 113 (8.6%) with at least one coinfection. Among the 113 co-infected cases, 79 (69.9%) cases were coinfected with dengue, followed by hepatitis E (*n* = 15, 13.2%), malaria (*n* = 9, 7.9%), typhoid (*n* = 9, 7.9%), and scrub typhus (*n* = 4, 3.5%). While all seven observational studies reported dengue as a co-infection, two studies have reported malaria [17, 18], followed by hepatitis E, typhoid, and scrub typhus being reported from single studies [Table 1].

**Table 1:** Distribution of observational studies of leptospirosis with other co-infection cases

Author/Year (reference number)	Location of the study	Sample size (*n* = 9031)	Leptospirosis positive cases (*n* = 1318)	Diagnosis of Leptospirosis	Total no. of patients with co-infections (*n* = 113)	Co-infections	Diagnosis of Co-infections
Dhanashree, B., 2021 [19]	Mangalore	811	119	ELISA	9	Dengue	IgM ELISA
Ravindar A, 2018 [20]	Chennai, Tamil Nadu	100	18	ELISA, PCR	4	Dengue	IgM ELISA, NS1Ag, PCR
Sachu A., 2018 [4]	Alappuzha, Kerala	2396	193	ELISA	33	Dengue	IgM ELISA
Chaudhry R, 2017 [17]	New Delhi, India	704	144	ELISA	19	Malaria (1) Scrub typhus (4) Dengue (5) Typhoid (9)	Peripheral blood smear, ICT, QBC assay IgM ELISA IgM ELISA Widal Test
Ananthi B, 2014 [21]	Chennai, Tamil Nadu	143	143	MAT	4	Dengue	IgM ELISA
Chaudhry R, 2013 [18]	New Delhi	1453	414	ELISA, PCR, MAT	27	Malaria (8)^o^ Dengue (7)*^o^ Hepatitis E (15)*^o^	Peripheral blood smear, ICT, QBC assay IgM ELISA IgM ELISA
Kumar A, 2012 [22]	Kochi, Kerala	3424	287	ELISA, MAT	17	Dengue	IgM ELISA

(* Leptospirosis triple infection: 1 case of dengue and hepatitis E

^o^Multiple infection of leptospirosis: 1 case of dengue, malaria, and hepatitis E)

**Fig. 3:**
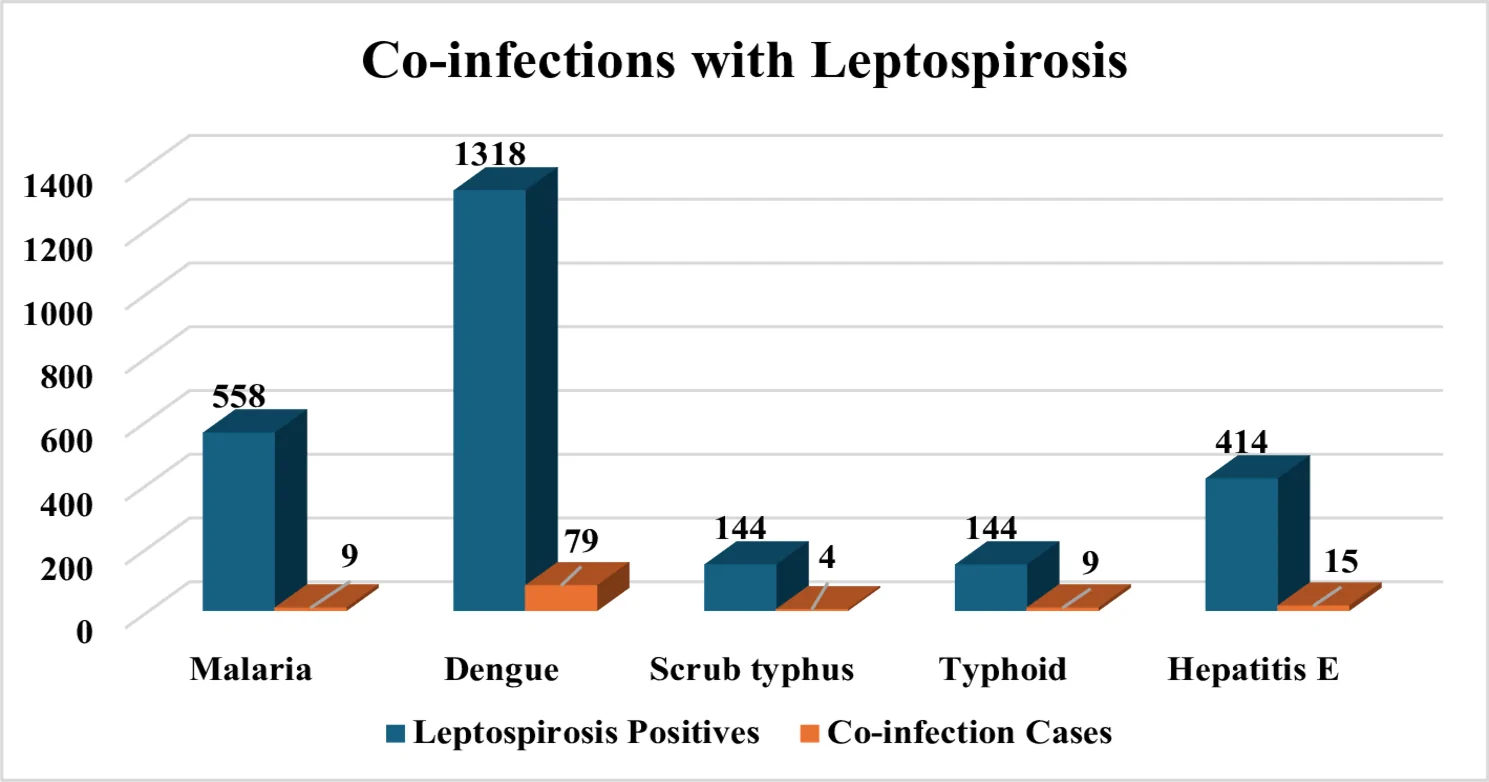
Distribution of leptospirosis co-infections among observational studies. (Blue bar represents the total number of leptospirosis cases screened for each co-infection; Orange bar denotes the number of co-infected cases)

Across seven observational studies, out of 1318 leptospirosis cases dengue was the most prevalent coinfection reported in 79 (5.9%) cases. In addition, a study of 414 leptospirosis cases [18] showed 15 (3.6%) cases of co-infection with hepatitis E, whereas another study of 144 leptospirosis cases [17] showed nine (6.2%) cases of co-infections with typhoid, followed by four (2.7%) cases of co-infection with scrub typhus. Notably, a total of 558 leptospirosis positives [17, 18] from two studies reported nine cases (1.6%) of co-infection with malaria [Table 1; Fig. 3].

The eight observational studies focusing on 3449 AFI patients reported 109 leptospirosis patients with 68 (62.3%) cases of coinfection. In these studies, the highest proportion of coinfection was found with scrub typhus (61.7%), followed by malaria (33.8%), dengue (20.5%), and typhoid (1.4%) [Table 2]. Table 3 presents case reports and case series of leptospirosis patients coinfected with COVID-19 (49%), malaria (20%), dengue (12.7%), scrub typhus (9%), typhoid (5.5%), and hepatitis E (5.5%) coinfections.

**Table 2:** Distribution of observation studies of AFI with leptospirosis co-infection [23]

Author/Year (reference number)	Location of the study	Sample size (*n* = 3449)	Leptospirosis positive cases (*n* = 109)	Diagnosis of Leptospirosis	Total no. of patients with co-infections (*n* = 68)	Co-infections	Diagnosis of Co-infections
Husain U, 2022 [24]	Lucknow, Uttar Pradesh	1743	23	ELISA	23	Scrub typhus	IgM ELISA
Kammili N, 2013 [25]	Secunderabad, Telangana	100	12	ELISA	3	Dengue	IgM ELISA, NS1Ag
Pandey S, 2022 [26]	Kolkata, West Bengal	152	1	ELISA	1	Malaria	Thin blood smear
Das BK, 2021 [27]	Bhubaneswar, Odisha	92	30	ELISA	6	Scrub typhus	IgM ELISA
Mandage R, 2020 [28]	New Delhi, India	66	21	PCR	21	Malaria (21)*^o^ Scrub typhus (2)*^o^ Dengue (9)*^o^	Peripheral blood smear, RDT, PCR PCR PCR
Borkakoty B, 2016 [29]	Dibrugarh, Assam	32	8	ELISA	8	Scrub typhus	IgM ELISA
Raina S, 2018 [30]	Kangra	1164	11	ELISA	3	Scrub typhus	IgM ELISA
Raja JM, 2016 [31]	Chennai, Tamil Nadu	100	3	ELISA	3	Malaria (1)* Dengue (2) Typhoid (1)*	Thin blood smear, RDT IgM ELISA Widal, Blood culture

(* Leptospirosis triple co-infection: I case of malaria and typhoid, 1 case of malaria and scrub typhus, 8 cases of malaria and dengue;

o Multiple infection of leptospirosis: 1 case of dengue, malaria, and scrub typhus)

**Table 3:** Distribution of case reports and case series of co-infection with leptospirosis

Author/Year (reference number)	Location of the study	Leptospirosis positive cases (*n* = 63)	Diagnosis of Leptospirosis	Total no. of patients with co-infections (*n* = 55)	Co-infections	Diagnosis of Co-infections
Shiddapur G, 2024 [32]	Pune	1	ELISA, PCR	1	Dengue	IgM ELISA, RT-PCR
Nazeera NN, 2024 [33]	Wayanad, Kerala	1	ELISA	1	Scrub typhus	IgM ELISA
Shiddapur G, 2024 [34]	Pune	1	ELISA	1	Influenza B	RT-PCR
Aina N, 2023 [35]	Tamil Nadu	2	ELISA	2	COVID-19 Typhoid fever	RT-PCR Widal test
Saboo K, 2023 [36]	Wardha	1	MAT	1	Malaria* Vibrio*	Thick Blood Smear CSF Culture
Hareshkumar CN, 2022 [37]	Pune Maharashtra	1	ELISA	1	Tuberculosis	CSF CBNAAT
Gupta N., 2022 [38]	Manipal	24	ELISA	24	COVID-19	RT-PCR
Singh Y, 2022 [39]	New Delhi	1	ELISA	1	COVID-19	RT-PCR
Singh A,2021 [40]	Wardha	1	ELISA	1	COVID-19	RT-PCR
Kharibam P., 2021[41]	Rishikesh, Uttarakhand	1	ELISA	1	Dengue* Hepatitis B*	IgM ELISA, NS1Ag HBsAg
Dev N., 2019 [42]	New Delhi	1	MAT, PCR	1	Scrub typhus	IgM ELISA, PCR
Mehta V, 2019 [43]	Rishikesh, Uttarakhand	1	ELISA	1	Scrub typhus	IgM ELISA
Negi, A., 2018 [44]	Lucknow, Uttar Pradesh	1	ELISA	1	Typhoid fever	Widal test
Samanta, 2014 [45]	Lucknow, Uttar Pradesh	1	MAT	1	Malaria	RDT
Singh R, 2014 [46]	Lucknow, Uttar Pradesh	1	ELISA	1	Dengue* Hepatitis E*	IgM ELISA IgM ELISA
Chopdekar, 2014 [47]	Mumbai	2	ELISA	2	Dengue	IgM ELISA
Sharma S, 2014 [48]	Port Blair, Andaman & Nicobar Islands	12	MAT	4	Malaria	RDT
Diwan, A.G., 2014 [49]	Pune, Maharashtra	1	ELISA	1	Scrub typhus	Weil-Felix test
Damodar T, 2013 [50]	Mangalore	1	ELISA, Dark Field Microscopy	1	Hepatitis B	HBsAg
Singh MP, 2012 [51]	Chandigarh	1	ELISA	1	Hepatitis E	RT-PCR
Mahajan S.K, 2012 [52]	Shimla	1	ELISA	1	Scrub typhus	IgM ELISA
Baliga, K.V., 2011 [53]	Pune, Maharashtra	2	MAT	2	Malaria	Peripheral Blood Smear
Gurjar, M., 2011 [54]	Lucknow, Uttar Pradesh	3	ELISA	3	Malaria	Peripheral Blood Smear
Behera, B., 2010 [55]	New Delhi	1	ELISA	1	Dengue* Hepatitis E*	IgM ELISA IgM ELISA

(* Leptospirosis triple co-infection: 1 case with malaria and vibrio; 1 case with dengue and hepatitis B; 2 cases with dengue and hepatitis E)

This review identified 16 cases of leptospirosis, with two coinfections. One case from Tamil Nadu involved typhoid and COVID-19 along with leptospirosis, while another case from Tamil Nadu featured malaria and typhoid fever combined with leptospirosis. There was also a case reported in Maharashtra of leptospirosis along with malaria and Vibrio. Additionally, one case of leptospirosis was associated with malaria and scrub typhus, and there were eight cases of leptospirosis combined with malaria and dengue from New Delhi. Furthermore, there were four instances of dengue accompanied by hepatitis along with leptospirosis reported from Lucknow, Uttarakhand, and New Delhi. Notably, we also observed two cases of multiple infections along with leptospirosis: one involving malaria, dengue, and scrub typhus, and another case comprising malaria, dengue, and hepatitis E, both from New Delhi.

#### Clinical manifestations

Clinical manifestations and laboratory features could be retrieved from 29 studies; 10 articles did not describe all clinical aspects of coinfections. These clinical features represent coinfected leptospirosis cases only, as included studies lacked comparable monoinfection data. Fever was the most common symptom, reported in 72.7% of patients, followed by headache (26.4%), gastrointestinal symptoms (included vomiting (24.7%), abdominal pain (23.9%), and diarrhea (9.9%)), dyspnea (14.8%), chills (14%), oliguria (10.4%), altered sensorium (4.8%), and cough (3.3%); lymphadenopathy and muscle tenderness were reported less commonly (0.8%) [Supplementary File 1 – Table S6]. Reported clinical signs included rashes (24.7%), icterus (17.3%), conjunctival suffusion (11.5%), yellowish discoloration of the eyes (4.1%), hemorrhage (2.4%), hepatomegaly (14.8%), and splenomegaly (9.9%) [Supplementary File 1 – Table S7].

Among retrieved laboratory parameters from the 29 articles, thrombocytopenia was most commonly seen (65.2%), followed by transaminitis (55.3%), increased creatinine level (47.9%), and leucocytosis (28.9%) [Supplementary File 1 – Table S8]. Several articles reported complications like acute kidney injury (9.9%), and premorbidities like diabetes mellitus (3.3%) and hypertension (2.4%) [Supplementary File 1 – Table S6].

#### Treatment details, critical care utilizations, and outcomes

Twenty-five included articles reported on treatment details [Supplementary File 1 – Table S10]. Crystalline penicillin was given to 56% of patients, ceftriaxone to 24%, doxycycline to 38.4%, and azithromycin to 6.4%.

Co-infected patients have utilized critical care support during hospitalization due to complications and multiorgan dysfunction, documented in thirteen case reports and one observational study. Among these, seven patients (2.9%) were admitted to the ICU, thirteen patients (5.5%) underwent dialysis, and nine patients (3.8%) required ventilator support. Eight studies documented the average hospital stay duration, which was found to be 15.8 days. [Supplementary File 1 – Table S9]. Data on leptospirosis mono-infection were unavailable to compare the critical care support between mono-infected and co-infected patients.

The reported mortality rates in leptospirosis patients with coinfections (12%) were higher compared to mortality rates in leptospirosis mono-infected patients (5.2%) [Supplementary File 1 – Table S9]. Coinfection was associated with increased mortality for patients compared to isolated leptospirosis infections.

### Risk of bias assessment

The methodological quality assessment of included prevalence studies for the possibility of bias in its design, conduct, and analysis has been performed. In our study, we have found that RoB is low to moderate and no study has shown a high RoB. [Supplementary file 2 – Figure S1.a, S1.b, and S1.c].

## Discussion

Leptospirosis is a re-emerging infectious disease of public health importance [56] Concurrent infections in leptospirosis patients may significantly impact diagnosis, disease severity, and patient outcomes. This review is the first to collate and evaluate available evidence on the entities and relevance of coinfections in leptospirosis patients.

In this study, a total of 236 cases of leptospirosis with other co-infections were observed. The highest number of co-infections was seen in New Delhi, followed by Kerala, Karnataka, Uttar Pradesh, Tamil Nadu, and Maharashtra. Another review on leptospirosis from Manipal [57] showed that most cases were reported from coastal belts like Gujarat, Tamil Nadu, Kerala, Karnataka, and Maharashtra. Coastal states such as Kerala, Karnataka, Tamil Nadu, and Maharashtra consistently reported high leptospirosis prevalence due to their warm, humid climates, frequent flooding, and proximity to water bodies, all of which favor the survival and transmission of *Leptospira* spp. These regions also experience high rainfall and have large populations engaged in agriculture and occupations with increased exposure to contaminated water, further elevating risk.

In this review, the co-infection with dengue (69.9%) was more prevalent than other infections. Similarly, in another study from Malaysia, 29.1% of leptospirosis patients were co-infected with dengue [58]. Leptospira co-infection with other acute febrile illnesses occurs due to overlapping ecological factors. Leptospires thrive in waterlogged fields with rodents, increasing the risk for outdoor workers. Similarly, areas with mostly man-made waterlogging become dengue hotspots, especially during and after the monsoon season when Aedes mosquito breeding peaks [59]. Our observations indicate that a majority of co-infected cases were reported during the monsoon season. While most cases involved dual-agent co-infections, nine cases of triple co-infections and two multiple infections were identified. Studies reported similar triple infections with Campylobacter and Salmonella typhi in Ecuador [60], and hepatitis A with Epstein-Barr Virus in Romania [61].

Distinguishing leptospirosis from other acute febrile illnesses is crucial for management. Without microbiological testing, accurate identification remains challenging [57]. During the first week, blood culture or PCR is preferred as a diagnostic method [62]. In the second week, urine PCR or culture becomes significant, though cost and resources limit their use. After the first week, serology is recommended. The gold standard Microscopic Agglutination Test (MAT), available at reference centers, requires live cultures of leptospires [62]. When MAT is unavailable, serological tests like IgM ELISA are employed for diagnostic purposes, due to their accessibility and rapid turnaround. A Chennai study [63] reported IgM ELISA’s sensitivity at 100% and specificity at 93%. Due to constraints of conventional serological methods, rapid tests for leptospirosis diagnosis have gained popularity [57]. However, these serological tests are prone to cross-reactivity with antigens from other pathogens, such as *Rickettsia* [64] and Hepatitis E [19], leading to false-positive results. Consequently, there remains the possibility that reported dual infections were misdiagnoses due to cross-reactivity, rather than true co-infections. This highlights the necessity for confirmatory testing using molecular methods, such as PCR or culture. The persistence of anti-*Leptospira* IgM antibodies from recent infection may contribute to serological cross-reactivity with other pathogens, potentially leading to diagnostic misinterpretation [65]. Heterogeneity in the number and type of diagnostic assays used across studies is an important factor that may influence the detection rate of coinfections. Broader testing panels in certain studies likely increased the probability of detecting multiple pathogens, whereas narrower panels in others may have led to underdetection.

*Leptospira spp*. is divided into multiple serovars, with icterohaemorrhagiae being most prevalent in Indian studies. A systematic review from Manipal [57] identified icterohaemorrhagiae, australis, autumnalis, grippotyphosa, canicola, pomona, and pyrogenes as common serovars across Indian sites. A Tamil Nadu study [63] sowed higher icterohaemorrhagiae frequency in rural areas due to increased exposure to contaminated water and livestock. Conversely, another study from Tamil Nadu [66] found Canicola as the predominant serovar, with a positivity rate of 43.7% by MAT. The prevalence of serovar icterohaemorrhagiae and Canicola reflects the diversity of leptospiral transmission in India.

Clinical symptoms range from asymptomatic to severe complications in 5–10% of cases, presenting as Weil’s disease with organ dysfunction, jaundice, hemorrhage, meningitis, and acute kidney injury requiring intensive care assistance [67]. In this review, the most exhibited clinical manifestation among co-infected patients was fever (72.7%), followed by headache (26.4%), vomiting, and rashes (24.7%). Laboratory parameters showed thrombocytopenia (65.2%), transaminitis (55.3%), and increased creatinine (47.9%). Conversely, a Taiwan study [23] showed icterus and splenomegaly as predominant manifestations (71%), followed by lymphadenopathy (57%), oliguria and elevated creatinine (43%).

Treatment details were sparse in some studies, highlighting variability in the reporting of treatment modalities for leptospirosis, especially with co-infections. Crystalline penicillin, ceftriaxone, doxycycline, and azithromycin were the main antibiotics used, reflecting adherence to guidelines that recommend penicillin and doxycycline as first-line therapies, with ceftriaxone and azithromycin as alternatives for severe cases or when tetracyclines are contraindicated. Treatment options for patients with co-infections were documented in 86% of studies; however, similar information was not available for cases of isolated leptospirosis infections, which restricts the ability to compare co-infected and mono-infected cases. The need for organ support, including dialysis and ventilator assistance, was mainly observed in patients with renal and multi-organ involvement, highlighting disease severity.

Importantly, mortality was substantially higher in co-infected patients (12%) compared to those with mono-infection (5.2%). A systematic review on leptospirosis mono-infection cases [57] showed a 11% mortality rate across India. In North Kerala, leptospirosis mortality was 6.03%, with pulmonary and neurological involvement linked to mortality [68] Another Kerala study reported 15% case fatality due to cardiac involvement and bleeding manifestations [69]. Studies from New Delhi, Lucknow, and Kerala confirm that multi-organ dysfunction, renal failure, and cardiac or neurological involvement predict poor outcomes. Coinfection led to increased mortality compared to mono-infections. Mortality in co-infected patients stems from delayed pathogen detection, where delayed diagnosis of one infection impedes timely intervention, compounded by the additive or synergistic effects of dual infections on multiple organ dysfunction, leading to higher mortality risk compared to mono-infections.

## Limitations of the study

Several constraints affected this review. The evidence base was predominantly composed of case reports; however, there was a scarcity of observational studies specifically addressing leptospirosis co-infections. Furthermore, most observational studies lacked comprehensive information on coinfected cases, limiting the ability to fully assess the burden and characteristics of coinfection. In this review, the diagnosis of dual infections were diagnosed using both serological and confirmatory tests, with serological methods being employed more commonly. Further, the observed association between coinfection and disease severity may be influenced by ascertainment bias. Also, stronger IgM responses in severe disease may increase cross-reactivity and lead to false-positive serological diagnoses.

## Conclusion

This study highlights the significant impact of leptospirosis co-infections in India, particularly in New Delhi, followed by Kerala, Karnataka, Uttar Pradesh, and Tamil Nadu. Our findings indicate that dengue is the most frequent co-infecting pathogen, followed by hepatitis E, malaria, typhoid, and scrub typhus. The overlap in clinical symptoms among these infections, including thrombocytopenia, icterus, and renal impairment, makes timely diagnosis and treatment challenging. Leptospirosis co-infection was associated with poorer outcomes for patients compared to isolated infections. Further investigations are necessary to gain insight into the co-infections associated with leptospirosis.

## Electronic Supplementary Material

Below is the link to the electronic supplementary material.

Supplementary Material 1

Supplementary Material 2

## Data Availability

No datasets were generated or analysed during the current study.

## Abbreviations

AFI: Acute Febrile Illness
ARDS: Acute Respiratory Distress Syndrome
SPHS: Severe Pulmonary Haemorrhage Syndrome
PRISMA-ScR: Preferred Reporting Items for Systematic Reviews and Meta-Analyses Extension for Scoping Reviews
PICOS: (Population (or Patient/Problem), Intervention (or Exposure), Comparison (or Control), Outcome(s), and Study design)
OSF: Open Science Framework
JBI RoB: Joanna Briggs Institute’s Risk of Bias
ELISA: Enzyme-Linked Immunosorbent Assay
MAT: Microscopic Agglutination Test
