# Supplementary material for: Leptospirosis and its co-infections in India: prevalence, geographic distribution, and clinical implications
Source: BMC Infect Dis. 2026 Jun 26;26:1653. doi: 10.1186/s12879-026-13642-7 (PMC13579849; doi:10.1186/s12879-026-13642-7)
Supplement: Supplementary file 1 [file 12879_2026_13642_MOESM1_ESM.docx]

**Supplementary File – 1**

**List of Tables:**

| Table S1 | Distribution of search strategies developed for each database |
| --- | --- |
| Table S2 | Distribution of Co-infection cases with Leptospirosis |
| Table S3 | Table 3: Distribution of studies reporting leptospirosis coinfection cases confirmed by non-serological tests |
| Table S4 | Distribution of diagnostic assays used for the targeted pathogens |
| Table S5 | Distribution of studies with targeted pathogens vs detected pathogens |
| Table S6 | Clinical symptoms in patients with leptospirosis co-infection |
| Table S7 | Physical examination findings in patients with leptospirosis co-infection |
| Table S8 | Laboratory parameters in leptospirosis co-infection |
| Table S9 | Critical care support utilization during hospitalization in leptospirosis co-infection |
| Table S10 | Antibiotic administered in patients with leptospirosis co-infection |

| **Database** | **Search strategies** | **No. of articles included (2010-2024)** |
| --- | --- | --- |
| PUBMED | ((((India) AND (leptospirosis)) OR (leptospiral infection)) OR (leptospira)) AND (co-infections) | 154 |
| SCOPUS | ((India) AND (leptospirosis) OR (leptospiral AND infection) OR (leptospira) AND (co-infection) OR (coinfection)) | 45 |
| EMBASE | (('India'/exp OR India) AND leptospirosis OR (leptospiral AND infection)) AND coinfection | 104 |
| WEB OF SCIENCE | India (All Fields) and leptospirosis (All Fields) or leptospiral infection (All Fields) and co-infections (All Fields) | 631 |
|  | Total articles retrieved | 934 |

**Table S1: Distribution of search strategies developed for each database**

**Table S2: Distribution of Co-infection cases with Leptospirosis**

| **Author/Year (reference number)** | **Location of the study** | **Leptospirosis positive cases (n=1553)** | **Diagnosis of Leptospirosis** | **Total no. of patients with co-infections (n=236)** | **Co-infections** | **Diagnosis of Co-infections** |
| --- | --- | --- | --- | --- | --- | --- |
| **Observational studies** | | | | | | |
| Kumar A, 2012 (26) | Kochi, Kerala | 287 | IgM ELISA,  MAT | 17 | Dengue | Dengue IgM ELISA |
| Ananthi B, 2014 (27) | Chennai,  Tamil Nadu | 143 | MAT | 4 | Dengue | Dengue IgM ELISA |
| Dhanashree, B., 2021 (19) | Mangalore | 119 | IgM ELISA | 9 | Dengue | Dengue IgM ELISA |
| Sachu A., 2018 (4) | Alappuzha, Kerala | 193 | IgM ELISA | 33 | Dengue | Dengue IgM ELISA |
| Chaudhry R, 2017 (28) | New Delhi, India | 144 | IgM ELISA | 19 | Malaria  Scrub typhus  Dengue  Typhoid | Peripheral blood smear, ICT, QBC assay  Scrub typhus IgM ELISA  Dengue IgM ELISA  Widal Test |
| Ravindar A, 2018 (29) | Chennai,  Tamil Nadu | 18 | IgM ELISA, PCR | 4 | Dengue | IgM ELISA, NS1Ag, Dengue RT-PCR |
| Chaudhry R, 2013 (30) | New Delhi | 414 | IgM ELISA, PCR, MAT | 27 | Malaria  Dengue  Hepatitis E | Peripheral blood smear, ICT, QBC assay  Dengue IgM ELISA  Anti- HEV IgM ELISA |
| Husain U, 2022 (37) | Lucknow,  Uttar Pradesh | 23 | IgM ELISA | 23 | Scrub typhus | Scrub typhus IgM ELISA |
| Kammili N, 2013 (35) | Secunderabad, Telangana | 12 | IgM ELISA | 3 | Dengue | Dengue IgM ELISA, NS1Ag |
| Pandey S, 2022 (38) | Kolkata,  West Bengal | 1 | IgM ELISA | 1 | Malaria | Peripheral blood smear |
| Das BK, 2021 (32) | Bhubaneswar, Odisha | 30 | IgM ELISA | 6 | Scrub typhus | Scrub typhus IgM ELISA |
| Mandage R, 2020 (36) | New Delhi, India | 21 | PCR | 21 | Malaria  Scrub typhus  Dengue | Peripheral Blood Smear, RDT, PCR  Scrub typhus PCR  Dengue RT-PCR |
| Borkakoty B, 2016 (33) | Dibrugarh, Assam | 8 | IgM ELISA | 8 | Scrub typhus | Scrub typhus IgM ELISA |
| Raina S, 2018 (31) | Kangra | 11 | IgM ELISA | 3 | Scrub typhus | Scrub typhus IgM ELISA |
| Raja JM, 2016 (34) | Chennai,  Tamil Nadu | 3 | IgM ELISA | 3 | Malaria  Dengue  Typhoid | Thin blood smear, RDT  IgM ELISA  Widal, Blood culture |
| **Case Reports** | | | | | | |
| Dev N., 2019 (44) | New Delhi | 1 | MAT,  PCR | 1 | Scrub typhus | Scrub typhus IgM ELISA, PCR |
| Singh R, 2014 (45) | Lucknow, Uttar Pradesh | 1 | IgM ELISA | 1 | Dengue Hepatitis E | Dengue IgM ELISA  Anti-HEV IgM ELISA |
| Mehta V, 2019 (46) | Rishikesh, Uttarakhand | 1 | IgM ELISA | 1 | Scrub typhus | Scrub typhus IgM ELISA |
| Hareshkumar CN, 2022 (47) | Pune Maharashtra | 1 | IgM ELISA | 1 | Tuberculosis | CSF CBNAAT |
| Nazeera NN, 2024 (48) | Wayanad, Kerala | 1 | IgM ELISA | 1 | Scrub typhus | Scrub typhus IgM ELISA |
| Negi, A., 2018 (49) | Lucknow,  Uttar Pradesh | 1 | IgM ELISA | 1 | Typhoid fever | Widal test |
| Diwan, A.G., 2014 (50) | Pune, Maharashtra | 1 | IgM ELISA | 1 | Scrub typhus | Weil-Felix test |
| Singh MP, 2012 (39) | Chandigarh | 1 | IgM ELISA | 1 | Hepatitis E | RT-PCR |
| Shiddapur G, 2024 (40) | Pune | 1 | IgM ELISA,  PCR | 1 | Dengue | Dengue IgM ELISA,  RT-PCR |
| Singh A,2021 (41) | Wardha | 1 | IgM ELISA | 1 | COVID-19 | COVID-19 RT-PCR |
| Saboo K, 2023 (42) | Wardha | 1 | MAT | 1 | Malaria*    Vibrio mimicus* | Peripheral Blood Smear  CSF Culture |
| Kharibam P., 2021(43) | Rishikesh, Uttarakhand | 1 | IgM ELISA | 1 | Dengue    Hepatitis B | Dengue IgM ELISA, NS1Ag  HBsAg |
| Samanta, 2014 (51) | Lucknow, Uttar Pradesh | 1 | MAT | 1 | Malaria | Malaria RDT |
| Chopdekar, 2014 (52) | Mumbai, Maharashtra | 2 | IgM ELISA | 2 | Dengue | Dengue IgM ELISA |
| Baliga, K.V., 2011 (53) | Mumbai, Maharashtra | 2 | MAT, *Leptospira* IgM ELISA | 2 | Malaria | Peripheral Blood Smear QBC |
| Behera, B., 2010 (54) | New Delhi | 1 | IgM ELISA | 1 | Dengue Hepatitis E | Dengue IgM ELISA  Anti-HEV IgM ELISA |
| Shiddapur G, 2024 (55) | Pune, Maharashtra | 1 | IgM ELISA | 1 | Influenza B | Influenza B RT-PCR |
| Singh Y, 2022 (56) | New Delhi | 1 | IgM ELISA | 1 | COVID-19 | COVID-19 RT-PCR |
| Damodar T, 2013 (59) | Mangalore | 1 | IgM ELISA,  Dark Field Microscopy | 1 | Hepatitis B | Anti-HAV IgM ELISA  RDT |
| Mahajan S.K, 2012 (58) | Shimla | 1 | IgM ELISA | 1 | Scrub typhus | Scrub typhus IgM ELISA |
| **Case Series** | | | | | | |
| Aina N, 2023 (59) | Tamil Nadu | 2 | IgM ELISA | 2 | COVID-19 Typhoid fever | COVID-19 RT-PCR  Widal test |
| Gupta N., 2022 (60) | Manipal | 24 | IgM ELISA | 24 | COVID-19 | COVID-19 RT-PCR |
| Gurjar, M., 2011 (61) | Lucknow,  Uttar Pradesh | 3 | IgM ELISA | 3 | Malaria | Peripheral Blood Smear |
| Sharma S, 2014 (62) | Port Blair, Andaman & Nicobar Islands | 12 | MAT | 8 | Malaria | Malaria RDT |

(RDT: Rapid Diagnostic Tests; PCR: Polymerase Chain Reaction; RT-PCR: Reverse Transcriptase Polymerase Chain Reaction; ICT: Immunochromatographic Test; QBC: Quantitative Buffy Coat)

**Table S3**: **Distribution of studies reporting leptospirosis coinfection cases confirmed by non-serological tests**

| **Author/Year (reference number)** | **Location of the study** | **Leptospirosis positive cases (n=1553)** | **Diagnosis of Leptospirosis** | **Total no. of patients with co-infections (n=236)** | **Co-infections** | **Diagnosis of Co-infections** |
| --- | --- | --- | --- | --- | --- | --- |
| Shiddapur G, 2024 (40) | Pune | 1 | IgM ELISA,  PCR | 1 | Dengue | IgM ELISA, RT-PCR |
| Saboo K, 2023 (42) | Wardha | 1 | MAT | 1 | Malaria*  Vibrio mimicus* | Thick Blood Smear  CSF Culture |
| Mandage R, 2020 (36) | New Delhi, India | 21 | PCR | 21 | Malaria  Scrub typhus  Dengue | Peripheral Blood Smear, RDT, PCR  PCR  RT-PCR |
| Dev N., 2019 (44) | New Delhi | 1 | MAT,  PCR | 1 | Scrub typhus | IgM ELISA, PCR |
| Ravindar A, 2018 (29) | Chennai,  Tamil Nadu | 18 | IgM ELISA, PCR | 4 | Dengue | IgM ELISA, NS1Ag, RT-PCR |
| Chaudhry R, 2013 (30) | New Delhi | 414 | IgM ELISA, PCR, MAT | 27 | Malaria  Dengue  Hepatitis E | Peripheral blood smear, ICT, QBC assay  IgM ELISA  IgM ELISA |
| Baliga, K.V., 2011 (53) | Pune, Maharashtra | 2 | MAT | 2 | Malaria | Peripheral Blood Smear |

(RDT: Rapid Diagnostic Tests; PCR: Polymerase Chain Reaction; RT-PCR: Reverse Transcriptase Polymerase Chain Reaction; ICT: Immunochromatographic Test; QBC: Quantitative Buffy Coat; MAT: Microscopic Agglutination Test)

| **Author/Year (reference number)**  **Table S4: Distribution of diagnostic assays used for the targeted pathogens** | **Pathogens/Infection targeted** | **Diagnostic assays used (no. of positives)** | **Total no. of diagnostic assays used** | **Co-infections** |
| --- | --- | --- | --- | --- |
| **Observational studies** | | | | |
| Kumar A, 2012 (26) | Leptospirosis  Dengue | *Leptospira* IgM ELISA (287)  MAT (16)  MAC ELISA for Dengue (17) | 3 | Dengue |
| Ananthi B, 2014 (27) | Leptospirosis  Dengue | MSAT & MAT (143)  Dengue IgM ELISA (4) | 3 | Dengue |
| Dhanashree, B., 2021 (19) | Leptospirosis  Dengue  Malaria  HIV  Hepatitis B, C, E  Typhoid  Scrub typhus | *Leptospira* IgM ELISA (119)  Dengue IgM ELISA (9)  Peripheral blood smear  Serology for HIV,  Hepatitis B, C, E  Widal test  Weil-Felix test | 9 | Dengue |
| Sachu A., 2018 (4) | Leptospirosis  Dengue | *Leptospira* IgM ELISA (193)  Dengue IgM ELISA (33) | 2 | Dengue |
| Chaudhry R, 2017 (28) | Leptospirosis  Dengue  Malaria  Enteric fever  Scrub typhus | *Leptospira* IgM ELISA (144)  Dengue IgM ELISA (5)  Peripheral Blood Smear (1)  ICT, QBC  Widal test (9)  Scrub typhus IgM ELISA | 7 | Malaria  Scrub typhus  Dengue  Typhoid |
| Ravindar A, 2018 (29) | Leptospirosis  Dengue | *Leptospira* IgM ELISA (8)  *Leptospira* PCR (10)  Dengue IgM (15) & IgG ELISA (8)  Dengue NS1Ag ELISA (22)  RT-PCR | 6 | Dengue |
| Chaudhry R, 2013 (30) | Leptospirosis  Dengue  Malaria  Hepatitis A, E Virus | *Leptospira* IgM ELISA (391)  MAT (50), *Leptospira* PCR (10)  Blood & Urine culture (5)  Dengue IgM ELISA (7)  Peripheral blood smear  ICT, QBC  IgM ELISA for Hepatitis A, E (15) | 11 | Malaria  Dengue  Hepatitis E |
| Husain U, 2022 (37) | Leptospirosis  Scrub typhus | *Leptospira* IgM ELISA (23)  Scrub typhus IgM ELISA (361) | 2 | Scrub typhus |
| Kammili N, 2013 (35) | Leptospirosis  Dengue  Chikungunya  Enteric fever  Malaria  Scrub typhus | *Leptospira* IgM ELISA (12)  Dengue IgM & IgG ELISA, Dengue NS1Ag (39)  Chikungunya MAC ELISA (4)  Widal test (1),  RDT – Malaria P.v/P.f (7)  Weil Felix (19),  Scrub typhus IgM (19) &  IgG ELISA, ICT | 11 | Dengue |
| Pandey S, 2022 (38) | Leptospirosis  Dengue  Chikungunya  Enteric fever  Malaria | *Leptospira* IgM ELISA (1)  Dengue IgM ELISA, Dengue NS1Ag  Chikungunya IgM ELISA  Typhoid IgM ELISA,  Blood culture  Peripheral Blood Smear (1) | 7 | Malaria |
| Das BK, 2021 (32) | Leptospirosis  Dengue  HSV 1 & 2  Epstein-Barr Virus  Cytomegalovirus  Japanese encephalitis  Parvo B19  Scrub typhus | *Leptospira* IgM ELISA (42)  Serology for Dengue (3), HSV 1 (4) & HSV 2 (3), EBV (22), CMV, JE (27), Parvo B19  PCR for Parvo B19  Scrub typhus IgM ELISA (11) | 10 | Scrub typhus |
| Mandage R, 2020 (36) | Leptospirosis  Dengue  Chikungunya  Scrub typhus  *P. falciparum, P. vivax, P. malaria, P. ovale, and P. knowlesi* | PCR for Leptospirosis (21),  Dengue (9),  Chikungunya,  Scrub typhus (2),  5 *Plasmodium spp.*  Peripheral Blood Smear (66) | 10 | Malaria  Scrub typhus  Dengue |
| Borkakoty B, 2016 (33) | Leptospirosis  Scrub typhus  Malaria | *Leptospira* IgM ELISA (8)  Scrub typhus IgM ELISA (32)  Peripheral Blood Smear | 3 | Scrub typhus |
| Raina S, 2018 (31) | Leptospirosis  Scrub typhus  Dengue  Chikungunya  Malaria | *Leptospira* IgM ELISA (11)  Scrub typhus IgM ELISA (262)  Dengue MAC ELISA, Dengue NS1Ag  Chikungunya IgM ELISA  Peripheral blood smear  Blood culture | 7 | Scrub typhus |
| Raja JM, 2016 (34) | Leptospirosis  Dengue  Malaria  Enteric fever | *Leptospira* IgM ELISA (3),  *Leptospira* RDT  Dengue IgM ELISA (12)  Peripheral Blood Smear (4), Malaria RDT  Widal test (4), Blood culture | 7 | Malaria  Dengue  Typhoid |
| **Case Reports** | | | | |
| Dev N., 2019 (44) | Leptospirosis  Scrub typhus | *Leptospira* PCR (1)  MAT (1)  Scrub typhus IgM ELISA (1)  Scrub typhus PCR (1) | 2 | Scrub typhus |
| Singh R, 2014 (45) | Leptospirosis  Dengue    Hepatitis E Virus  Malaria, Enteric fever, HIV, Hepatitis A, B, C Virus | *Leptospira* IgM ELISA (1)  Dengue IgM ELISA (1),  Dengue NS1Ag  Anti-HEV IgM (1)  [Malaria, Enteric fever, HIV, Hepatitis A, B, C (NP*)] | 4 | Dengue Hepatitis E |
| Mehta V, 2019 (46) | Leptospirosis  Scrub typhus | *Leptospira* IgM ELISA (1)  ICT for scrub typhus (1) | 2 | Scrub typhus |
| Hareshkumar CN, 2022 (47) | Leptospirosis  *Mycobacterium tuberculosis*  HIV, COVID-19  Dengue  Hepatitis B & C Virus | *Leptospira* RDT (1)  CBNAAT (CSF) (1)  RT-PCR for HIV and COVID-19  Dengue IgM ELISA, Dengue NS1Ag  HBsAg, Anti-HCV IgM | 8 | Tuberculosis |
| Nazeera NN, 2024 (48) | Leptospirosis  Dengue  Malaria  Scrub typhus | *Leptospira* IgM ELISA (1)  Dengue IgM ELISA, Dengue NS1Ag  Peripheral blood smear  Scrub typhus IgM ELISA (1)  Blood & Urine culture | 7 | Scrub typhus |
| Negi, A., 2018 (49) | Leptospirosis  Hepatitis A Virus  Hepatitis E Virus  Tuberculosis  Enteric fever | *Leptospira* IgM ELISA (1)  Anti-HAV IgM  Anti-HEV IgM  Acid-Fast Bacilli Microscopy  Widal test (1)  Blood & Urine culture | 7 | Typhoid fever |
| Diwan, A.G., 2014 (50) | Leptospirosis  Dengue  Malaria  Scrub typhus  HBV  HIV, HAV, HEV | *Leptospira* IgM ELISA (1)  Dengue IgM & IgG ELISA, Dengue NS1Ag  Peripheral blood smear, Malaria RDT  Weil-Felix test (1)  HBsAg  Serology for HIV, HAV, HEV | 11 | Scrub typhus |
| Singh MP, 2012 (39) | Leptospirosis  Dengue  Hepatitis A, B, C, E Virus  Epstein-Barr Virus  CMV  HSV | *Leptospira* IgM ELISA (1)  Dengue IgM ELISA  Anti-HAV IgM,  HBsAg, HBcAg  Anti-HCV IgM, HCV RT-PCR  Anti-HEV IgM, HEV RT-PCR (1)  Anti-VCA IgM  Anti-CMV IgM  Anti-HSV IgM | 12 | Hepatitis E |
| Shiddapur G, 2024 (40) | Leptospirosis  Dengue  Malaria  Typhoid fever  Rickettsia  COVID-19 | *Leptospira* IgM ELISA (1), *Leptospira* rt-PCR (1)  Dengue RT-PCR (2), Dengue NS1Ag,  Dengue IgM (1) & IgG ELISA  Malaria RDT  Widal test  Weil-Felix  COVID RT-PCR | 10 | Dengue |
| Singh A,2021 (41) | Leptospirosis  COVID-19  Scrub typhus  Dengue  Malaria  Hepatitis A, B, C, E Virus  CMV, EBV, HIV | *Leptospira* IgM ELISA (1)  COVID RT-PCR (1)  [Scrub typhus, Dengue, Malaria (NP*)]  Serology for Hepatitis A, B, C, E Virus, CMV, EBV, HIV  Blood & Urine Culture | 11 | COVID-19 |
| Saboo K, 2023 (42) | Leptospirosis  Malaria  Meningitis  HIV | MAT (1)  Peripheral blood smear (1)  CSF culture (1),  [HIV (NP*)]  Blood, Sputum, & Urine culture | 6 | Malaria  Vibrio mimicus |
| Kharibam P., 2021(43) | Leptospirosis  Dengue  HBV | *Leptospira* IgM ELISA (1)  Dengue IgM ELISA (1), Dengue NS1Ag (1)  HBsAg (1), HBeAg, HBV RNA | 6 | Dengue  Hepatitis B |
| Samanta, 2014 (51) | Leptospirosis  Malaria  Scrub typhus  Enteric fever  Dengue | MAT (1)  Malaria RDT (1)  Weil-Felix test  TyphiDot RDT  Dengue IgM ELISA  Blood & Urine culture | 7 | Malaria |
| Chopdekar, 2014 (52) | Leptospirosis  Malaria  Typhoid  Dengue  Hepatitis A, B, E Virus | *Leptospira* IgM ELISA (2)  Peripheral Blood Smear  Widal test  Dengue MAC ELISA (2)  Anti-HAV IgM, HBsAg,  Anti-HEV IgM | 7 | Dengue |
| Baliga, K.V., 2011 (53) | Leptospirosis  Malaria  Dengue  Typhoid  Scrub typhus  Brucellosis | MAT (2), Dark Field Microscopy, *Leptospira* IgM ELISA  Peripheral blood smear (1), QBC (1), ICT  Dengue IgG ELISA  Widal test  Weil-Felix test  Brucella IgM ELISA | 10 | Malaria |
| Behera, B., 2010 (54) | Leptospirosis  Malaria  Enteric fever  Dengue  HBV, HCV, HEV | *Leptospira* IgM ELISA (1)  Peripheral blood smear  Widal test  Dengue IgM ELISA (1)  HBsAg, Anti-HCV IgM, Anti-HEV IgM (1)  Blood & Urine culture | 9 | Dengue Hepatitis E |
| Shiddapur G, 2024 (55) | Leptospirosis  Influenza B Virus | *Leptospira* IgM ELISA (1)  Influenza RT-PCR (1) | 2 | Influenza B |
| Singh Y, 2022 (56) | Leptospirosis  COVID-19 | *Leptospira* IgM ELISA (1)  COVID RT-PCR (1) | 2 | COVID-19 |
| Damodar T, 2013 (59) | Leptospirosis  Malaria  Dengue  HBV  Typhoid fever  Scrub typhus | *Leptospira* IgM ELISA (1), Dark Field Microscopy (1)  Peripheral blood smear  Dengue IgM ELISA  HBsAg (1)  Widal test  Weil-Felix test | 7 | Hepatitis B |
| Mahajan S.K, 2012 (58) | Leptospirosis  Malaria  Typhoid fever  Scrub typhus | *Leptospira* IgM ELISA (1)  Peripheral blood smear  Widal test  Scrub typhus IgM ELISA (1)  Blood & Urine culture | 6 | Scrub typhus |
| **Case Series** | | | | |
| Aina N, 2023 (59) | Leptospirosis  Enteric fever  COVID-19  Scrub typhus  Dengue  Tuberculosis, H1N1 Influenza | *Leptospira* IgM ELISA (2)  Widal test (3), Blood (1) & Bone marrow (1) culture  COVID RT-PCR (1)  Scrub typhus IgM ELISA (2)  Dengue IgM ELISA (1)  [Tuberculosis, H1N1 (NP*)]  Urine culture | 8 | COVID-19  Typhoid fever |
| Gupta N., 2022 (60) | Leptospirosis  COVID-19 | *Leptospira* IgM ELISA (1)  COVID RT-PCR (1) | 2 | COVID-19 |
| Gurjar, M., 2011 (61) | Leptospirosis  Malaria  Dengue | *Leptospira* IgM ELISA (3)  Peripheral Blood Smear(3), ICT (1)  Dengue IgM ELISA | 4 | Malaria |
| Sharma S, 2014 (62) | Leptospirosis  Malaria | MAT (12)  Malaria ICT (94) | 2 | Malaria |

(RDT: Rapid Diagnostic Tests; PCR: Polymerase Chain Reaction; RT-PCR: Reverse Transcriptase Polymerase Chain Reaction; ICT: Immunochromatographic Test; QBC: Quantitative Buffy Coat; MAC ELISA: IgM Antibody Capture ELISA; MAT: Microscopic Agglutination Test)

(* NP: Not provided)

| **Author/Year (reference number)**  **Table S5: Distribution of studies with targeted pathogens vs detected pathogens** | **Pathogens/Infection targeted** | **No. of pathogens targeted** | **Pathogen/Infection detected (n)** | **Total number of pathogens detected** |
| --- | --- | --- | --- | --- |
| **Observational Studies** | | | | |
| Kumar A, 2012 (26) | Leptospirosis  Dengue | 2 | Leptospirosis (287)  Dengue (239) | 2 |
| Ananthi B, 2014 (27) | Leptospira  Dengue | 2 | Leptospira (143)  Dengue (4) | 2 |
| Dhanashree, B., 2021 (19) | Leptospirosis  Dengue  Malaria  HIV  Hepatitis B, C, E  Typhoid  Scrub typhus | 9 | Leptospirosis (119)  Dengue (382) | 2 |
| Sachu A., 2018 (4) | Leptospirosis  Dengue | 2 | Leptospirosis (193)  Dengue (350) | 33 |
| Chaudhry R, 2017 (28) | Leptospirosis  Dengue  Malaria  Enteric fever  Scrub typhus | 5 | Leptospirosis (144)  Dengue (5)  Malaria (1)  Enteric fever (9)  Scrub typhus (4) | 5 |
| Ravindar A, 2018 (29) | Leptospirosis  Dengue | 2 | Leptospirosis (18)  Dengue (29) | 2 |
| Chaudhry R, 2013 (30) | Leptospirosis  Dengue  Malaria  Hepatitis A, E Virus | 5 | Leptospirosis (414)  Dengue (5)  Malaria (70  Hepatitis E (13) Virus | 4 |
| Husain U, 2022 (37) | Leptospirosis  Scrub typhus | 2 | Leptospirosis (23)  Scrub typhus (361) | 2 |
| Kammili N, 2013 (35) | Leptospirosis  Dengue  Chikungunya  Enteric fever  Malaria  Scrub typhus | 6 | Leptospirosis (9)  Dengue (39)  Enteric fever (1)  Malaria (7)  Scrub typhus (19) | 5 |
| Pandey S, 2022 (38) | Leptospirosis  Dengue  Chikungunya  Enteric fever  Malaria | 5 | Leptospirosis (1)  Dengue (1)  Chikungunya (1)  Enteric fever (1)  Malaria (125) | 5 |
| Das BK, 2021 (32) | Leptospirosis  Dengue  HSV 1 & 2  Epstein-Barr Virus  Cytomegalovirus  Japanese encephalitis  Parvo B19  Scrub typhus | 9 | Leptospirosis (30)  Dengue (3)  HSV 1 (4) & 2 (3)  Epstein-Barr Virus (22)  Cytomegalovirus (10)  Japanese encephalitis (27)  Scrub typhus (11) | 8 |
| Mandage R, 2020 (36) | Leptospirosis  Dengue  Chikungunya  Scrub typhus  *P. falciparum, P. vivax, P. malaria, P. ovale, and P. knowlesi* | 9 | Leptospirosis (11)  Scrub typhus (5)  *P. falciparum* (23)*, P. vivax* (50)*, and P. knowlesi* (12) | 5 |
| Borkakoty B, 2016 (33) | Leptospirosis  Scrub typhus  Malaria | 3 | Leptospirosis (8)  Scrub typhus (30) | 2 |
| Raina S, 2018 (31) | Leptospirosis  Scrub typhus  Dengue  Chikungunya  Malaria | 5 | Leptospirosis (8)  Scrub typhus (262)  Dengue (91)  Chikungunya (7)  Malaria (2) | 3 |
| Raja JM, 2016 (34) | Leptospirosis  Dengue  Malaria  Enteric fever | 4 | Leptospirosis (3)  Dengue (12)  Malaria (4)  Enteric fever (4) | 4 |
| **Case Reports** | | | | |
| Dev N., 2019 (44) | Leptospirosis  Scrub typhus | 2 | Leptospirosis (1)  Scrub typhus (1) | 2 |
| Singh R, 2014 (45) | Leptospirosis  Dengue  Hepatitis E Virus  Malaria, Enteric fever, HIV, Hepatitis A, B, C Virus | 9 | Leptospirosis (1)  Dengue (1)  Hepatitis E Virus (1) | 3 |
| Mehta V, 2019 (46) | Leptospirosis  Scrub typhus | 2 | Leptospirosis (1)  Scrub typhus (1) | 2 |
| Hareshkumar CN, 2022 (47) | Leptospirosis  *Mycobacterium tuberculosis*  HIV, COVID-19  Dengue  Hepatitis B & C Virus | 7 | Leptospirosis (1)  *Mycobacterium tuberculosis* (1) | 2 |
| Nazeera NN, 2024 (48) | Leptospirosis  Dengue  Malaria  Scrub typhus | 4 | Leptospirosis (1)  Scrub typhus (1) | 2 |
| Negi, A., 2018 (49) | Leptospirosis  Hepatitis A Virus  Hepatitis E Virus  Tuberculosis  Enteric fever | 5 | Leptospirosis (1)  Enteric fever (1) | 2 |
| Diwan, A.G., 2014 (50) | Leptospirosis  Dengue  Malaria  Scrub typhus  HBV  HIV, HAV, HEV | 8 | Leptospirosis (1)  Scrub typhus (1) | 2 |
| Singh MP, 2012 (39) | Leptospirosis  Dengue  Hepatitis A, B, C, E Virus  Epstein-Barr Virus  CMV  HSV | 9 | Leptospirosis (1)  Hepatitis E (1) Virus | 2 |
| Shiddapur G, 2024 (40) | Leptospirosis  Dengue  Malaria  Typhoid fever  Rickettsia  COVID-19 | 6 | Leptospirosis (1)  Dengue (1 | 2 |
| Singh A,2021 (41) | Leptospirosis  COVID-19  Scrub typhus  Dengue  Malaria  Hepatitis A, B, C, E Virus  CMV, EBV, HIV | 12 | Leptospirosis (1)  COVID-19 (1 | 2 |
| Saboo K, 2023 (42) | Leptospirosis  Malaria  Meningitis  HIV | 4 | Leptospirosis (1)  Malaria (1)  Meningitis (1) | 3 |
| Kharibam P., 2021(43) | Leptospirosis  Dengue  HBV | 3 | Leptospirosis (1)  Dengue (1)  HBV (1) | 3 |
| Samanta, 2014 (51) | Leptospirosis  Malaria  Scrub typhus  Enteric fever  Dengue | 5 | Leptospirosis (1)  Malaria (1) | 2 |
| Chopdekar, 2014 (52) | Leptospirosis  Malaria  Typhoid  Dengue  Hepatitis A, B, E Virus | **7** | Leptospirosis (1)  Dengue (1 | 2 |
| Baliga, K.V., 2011 (53) | Leptospirosis  Malaria  Dengue  Typhoid  Scrub typhus  Brucellosis | 6 | Leptospirosis (2)  Malaria (2) | 2 |
| Behera, B., 2010 (54) | Leptospirosis  Malaria  Enteric fever  Dengue  HBV, HCV, HEV | 7 | Leptospirosis (1)  Dengue (1)  Hepatitis E Virus (1) | 3 |
| Shiddapur G, 2024 (55) | Leptospirosis  Influenza B Virus | 2 | Leptospirosis (1)  Influenza B Virus (1) | 2 |
| Singh Y, 2022 (56) | Leptospirosis  COVID-19 | 2 | Leptospirosis (1)  COVID-19 (1) | 2 |
| Damodar T, 2013 (59) | Leptospirosis  Malaria  Dengue  HBV  Typhoid fever  Scrub typhus | 6 | Leptospirosis (1)  Hepatitis B Virus (1) | 2 |
| Mahajan S.K, 2012 (58) | Leptospirosis  Malaria  Typhoid fever  Scrub typhus | 4 | Leptospirosis (1)  Scrub typhus (1) | 2 |
| **Case Series** | | | | |
| Aina N, 2023 (59) | Leptospirosis  Enteric fever  COVID-19  Scrub typhus  Dengue  Tuberculosis, H1N1 Influenza | 7 | Leptospirosis (2)  Enteric fever (2)  COVID-19 (1)  Scrub typhus (2)  Dengue (1) | 5 |
| Gupta N., 2022 (60) | Leptospirosis  COVID-19 | 2 | Leptospirosis (87)  COVID-19 (50) | 2 |
| Gurjar, M., 2011 (61) | Leptospirosis  Malaria  Dengue | 3 | Leptospirosis (3)  Malaria (3) | 2 |
| Sharma S, 2014 (62) | Leptospirosis  Malaria | 2 | Leptospirosis (12)  Malaria (94) | 2 |

**Table S6: Clinical symptoms in patients with leptospirosis coinfection**

| **Author/Year** | **Leptospirosis positive cases (n=879)** | **No. of patients with Co-infections (n=121)** | **Fever** | **Chills** | **Cough** | **Headache** | **Vomiting** | **Abdominal pain** | **Diarrhea** | **Dyspnea** | **Oliguria** | **Altered sensorium** | **Lymphadenopathy** | **AKI** | **DM** | **HTN** |
| --- | --- | --- | --- | --- | --- | --- | --- | --- | --- | --- | --- | --- | --- | --- | --- | --- |
| Shiddapur, G, 2024 | 1 | 1 | 1 | 1 | - | - | - | - | - | - | - | 1 | - | - | - | - |
| Nazeera NN, 2024 | 1 | 1 | 1 | - | - | 1 | - | - | - | - | 1 | - | - | - | - | - |
| Shiddapur, G, 2024 | 1 | 1 | 1 | - | 1 | 1 | - | - | - | 1 | 1 | - | - | - | - | - |
| Aina, N, 2023 | 2 | 2 | 2 | - | - | 1 | 2 | 1 | 1 | - | - | - | - | - | - | - |
| Saboo K, 2023 | 1 | 1 | 1 | - | - | 1 | - | - | - | - | - | 1 | - | - | - | - |
| Hareshkumar CN, 2022 | 1 | 1 | 1 | - | - | - | - | - | - | - | - | 1 | - | 1 | 1 | 1 |
| Gupta, N., 2022 | 87 | 24 | 24 | - | - | - | - | 11 | 7 | 16 | - | - | - | - | - | - |
| Singh Y, 2022 | 1 | 1 | 1 | - | 1 | - | - | - | - | 1 | - | - | - | 1 | 1 | 1 |
| Singh, A,2021 | 1 | 1 | 1 | - | - | - | - | - | - | - | 1 | - | - | - | - | - |
| Dhanashree, B., 2021 | 119 | 9 | 9 | - | - | 1 | - | - | - | - | - | - | - | 2 | 1 | - |
| Kharibam, P., 2021 | 1 | 1 | 1 | 1 | 1 | - | 1 | 1 | - | - | 1 | - | - | 1 | - | - |
| Dev, N., 2019 | 1 | 1 | 1 | - | - | - | - | - | - | - | - | - | - | - | - | - |
| Mehta. V, 2019 | 1 | 1 | 1 | 1 | - | - | 1 | - | 1 | - | 1 | 1 | - | 1 | - | - |
| Negi, A., 2018 | 1 | 1 | 1 | 1 | - | - | 1 | 1 | 1 | - | - | - | - | - | - | - |
| Sachu, A., 2018 | 193 | 33 | - | - | - | - | 14 | 10 | - | - | - | - | - | - | - | - |
| Raina S, 2018 | 11 | 3 | 3 | 3 | - | 3 | - | 2 | - | - | - | - | - | - | - | - |
| Sharma S, 2014 | 12 | 4 | 4 | 4 | - | 4 | 4 | - | - | - | - | - | - | - | - | - |
| Diwan, A.G., 2014 | 1 | 1 | 1 | 1 | - | 1 | 1 | - | - | - | - | - | - | 1 | - | - |
| Samanta, 2014. | 1 | 1 | 1 | - | - | - | - | - | - | - | 1 | 1 | - | 1 | - | - |
| Singh R, 2014 | 1 | 1 | 1 | - | - | - | 1 | - | - | - | 1 | - | - | 1 | - | - |
| Chopdekar, 2014 | 2 | 2 | 2 | 1 | - | - | 1 | 1 | 1 | - | 1 | - | - | - | - | - |
| Ananthi, B, 2014 | 143 | 4 | 4 | - | - | - | - | - | - | - | - | - | - | - | - | - |
| Damodar T, 2013 | 1 | 1 | 1 | 1 | 1 | - | - | - | - | - | 1 | - | - | - | - | - |
| Singh, MP, 2012 | 1 | 1 | 1 | - | - | - | - | 1 | - | - | - | - | - | - | - | - |
| Mahajan S.K, 2012 | 1 | 1 | 1 | - | - | - | - | - | - | - | - | - | 1 | 1 | - | - |
| Kumar, A, 2012 | 287 | 17 | 17 | - | - | 17 | - | - | - | - | - | - | - | - | - | - |
| Baliga, K.V., 2011 | 1 | 1 | 2 | 1 | - | 2 | 1 | - | - | - | - | - | - | 1 | - | - |
| Gurjar, M., 2011 | 3 | 3 | 3 | 2 | - | - | 3 | 1 | 1 | - | 3 | 1 | - | - | 1 | 1 |
| Behera, B., 2010 | 1 | 1 | 1 | - | - | - | - | - | - | - | 1 | - | - | 1 | - | - |
| Total | 879 | 121 | 88 | 17 | 4 | 32 | 30 | 29 | 12 | 18 | 13 | 6 | 1 | 12 | 4 | 3 |

**Table S7: Physical examination findings in patients with leptospirosis co-infection**

| **Author/Year** | **Location of the study** | **Leptospirosis positive cases** | **Patients with Co-infections** | **Yellowish eyes** | **Rashes** | **Icterus** | **Redness of eyes** | **Muscle tenderness** | **Hemorrhage** | **Hepatomegaly** | **Splenomegaly** |
| --- | --- | --- | --- | --- | --- | --- | --- | --- | --- | --- | --- |
| Shiddapur, G, 2024 | Pune | 1 | 1 | - | - | - | - | - | - | 1 | - |
| Nazeera NN, 2024 | Wayanad, Kerala | 1 | 1 | - | - | - | - | - | - | - | - |
| Shiddapur G, 2024 | Pune | 1 | 1 | - | - | - | - | - | - | - | - |
| Aina, N, 2023 | Tamil Nadu | 2 | 2 | - | - | - | - | - | - | 1 | - |
| Saboo K, 2023 | Wardha | 1 | 1 | - | - | - | - | - | - | - | - |
| Hareshkumar CN, 2022 | Pune Maharashtra | 1 | 1 | 1 | - | - | - | - | - | 1 | - |
| Gupta, N., 2022 | Manipal | 87 | 24 | - | 2 | - | - | - | - | 1 | 1 |
| Singh Y, 2022 | New Delhi | 1 | 1 | - | - | 1 | - | - | - | - | - |
| Singh, A,2021 | Wardha | 1 | 1 | - | - | - | - | - | - | 1 | 1 |
| Dhanashree, B., 2021 | Mangalore | 119 | 9 | - | 9 | 1 | - | - | - | - | - |
| Kharibam, P., 2021 | Rishikesh, Uttarakhand | 1 | 1 | 1 | - | 1 | 1 | - | - | 1 | 1 |
| Raina S, 2018 | Kangra, | 11 | 3 | - | 1 | 3 | - | - | - | 3 | 2 |
| Dev, N., 2019 | New Delhi | 1 | 1 | 1 | - | 1 | - | - | - | 1 | - |
| Mehta. V, 2019 | Rishikesh, Uttarakhand | 1 | 1 | - | - | 1 | - | - | - | 1 | - |
| Negi, A., 2018 | Lucknow, Uttar Pradesh | 1 | 1 | - | - | 1 | - | - | - | - | - |
| Samanta, 2014. | Lucknow, Uttar Pradesh | 1 | 1 | - | - | 1 | 1 | - | 1 | 1 | 1 |
| Singh R, 2014 | Lucknow, Uttar Pradesh | 1 | 1 | - | - | 1 | - | - | - | - | - |
| Chopdekar, 2014 | Mumbai | 2 | 2 | - | - | - | - | - | - | - | 2 |
| Ananthi, B, 2014 | Chennai, Tamil Nadu | 143 | 4 | - | - | - | - | - | 1 | 1 | 1 |
| Sharma S, 2014 | Port Blair | 12 | 4 | - | - | 4 | - | - | - | - | - |
| Diwan, A.G., 2014 | Pune, Maharashtra | 1 | 1 | - | - | - | - | - | - | 1 | - |
| Damodar T, 2013 | Mangalore | 1 | 1 | - | - | 1 | - | - | 1 | - | - |
| Singh, MP, 2012 | Chandigarh | 1 | 1 | 1 | - | 1 | - | - | - | 1 | - |
| Mahajan S.K, 2012 | Shimla | 1 | 1 | - | 1 | - | - | 1 | - | - | 1 |
| Kumar, A, 2012 | Kochi, Kerala | 287 | 17 | - | Yes* | - | Yes* | - | - | - | - |
| Baliga, K.V., 2011 | Pune, Maharashtra | 1 | 1 | - | - | 2 | - | - | - | 2 | 2 |
| Gurjar, M., 2011 | Lucknow, Uttar Pradesh | 3 | 3 | 1 | - | - | - | - | - | 1 | - |
| Behera, B., 2010 | New Delhi | 1 | 1 | - | - | 1 | - | - | - | - | - |

[*Few patients were presented with clinical symptoms, but the number of patients has not been documented in the included study.]

**Table S8: Laboratory parameters in leptospirosis co-infection**

| **Author/Year** | **Location of the study** | **Leptospirosis positive cases** | **Patients with Co-infections** | **Thrombocytopenia** | **Transaminitis** | **Increased creatinine** | **Leucocytosis** |
| --- | --- | --- | --- | --- | --- | --- | --- |
| Shiddapur, G, 2024 | Pune | 1 | 1 | 1 | - | - | - |
| Nazeera NN, 2024 | Wayanad, Kerala | 1 | 1 | 1 | - | 1 |  |
| Shiddapur G, 2024 | Pune | 1 | 1 | 1 | - | - | 1 |
| Aina, N, 2023 | Tamil Nadu | 2 | 2 | - | 2 | - | - |
| Saboo K, 2023 | Wardha | 1 | 1 | 1 | 1 | - | - |
| Hareshkumar CN, 2022 | Pune Maharashtra | 1 | 1 | 1 | - | 1 | 1 |
| Gupta, N., 2022 | Manipal | 87 | 24 | 22 | 15 | 19 | 11 |
| Singh Y, 2022 | New Delhi | 1 | 1 | 1 | - | - | - |
| Singh, A,2021 | Wardha | 1 | 1 | 1 | - | 1 | - |
| Kharibam, P., 2021 | Rishikesh, Uttarakhand | 1 | 1 | 1 | - | 1 | - |
| Dhanashree, B., 2021 | Mangalore | 119 | 9 | 3 | 9 | 9 | 9 |
| Dev, N., 2019 | New Delhi | 1 | 1 | - | - | - | - |
| Mehta. V, 2019 | Rishikesh, Uttarakhand | 1 | 1 | 1 | - | - | - |
| Negi, A., 2018 | Lucknow, Uttar Pradesh | 1 | 1 | 1 | - | - | - |
| Sachu, A., 2018 | Alappuzha, Kerala | 193 | 33 | 24 | 20 | 5 | 6 |
| Raina S, 2018 | Kangra | 11 | 3 | - | - | - | - |
| Samanta, 2014. | Lucknow, Uttar Pradesh | 1 | 1 | 1 | - | 1 | - |
| Singh R, 2014 | Lucknow, Uttar Pradesh | 1 | 1 | 1 | 1 | 1 | - |
| Ananthi B, 2014 | Chennai, Tamil Nadu | 143 | 4 | 3 | - | - | - |
| Chopdekar, 2014 | Mumbai | 2 | 2 | 1 | 1 | - | - |
| Diwan, A.G., 2014 | Pune, Maharashtra | 1 | 1 | - | - | - | - |
| Damodar T, 2013 | Mangalore | 1 | 1 | 1 | 1 | 1 | - |
| Singh, MP, 2012 | Chandigarh | 1 | 1 | - | 1 | - | - |
| Mahajan S.K, 2012 | Shimla | 1 | 1 | 1 | - | - | - |
| Kumar A, 2012 | Kochi, Kerala | 287 | 17 | 11 | 16 | 16 | 6 |
| Baliga, K.V., 2011 | Pune, Maharashtra | 1 | 1 | 1 | - | - | 1 |
| Gurjar, M., 2011 | Lucknow, Uttar Pradesh | 3 | 3 | - | - | 2 | - |
| Behera, B., 2010 | New Delhi | 1 | 1 | - | - | - | - |

**Table S9: Critical care support utilization during hospitalization in leptospirosis co-infection**

| **Author/Year** | **Location of the study** | **Leptospirosis positive cases** | **Patients with Co-infections** | **Average duration of ICU Stay in Co-infections** | **Dialysis in Co-infected patients** | **Ventilator support in Co-infected patients** | **Average duration of Hospital stay in Co-infected patients** | **Mortality in Co-infected patients** | **Mortality in Leptospirosis mono-infection** |
| --- | --- | --- | --- | --- | --- | --- | --- | --- | --- |
| Siddapur G, 2024 | Pune | 1 | 1 | - | - | - | 7 | - | - |
| Nazeera NN, 2024 | Wayanad, Kerala | 1 | 1 | - | 1 | 1 | - | - | - |
| Singh Y, 2022 | New Delhi | 1 | 1 | 1 | - | - | - | 1 | - |
| Gupta N, 2022 | Manipal | 87 | 24 | - | - | - | - | 5 | 4 |
| Hareshkumar CN, 2022 | Pune Maharashtra | 1 | 1 | - | 1 | - | - | - | - |
| Dhanashree, B., 2021 | Mangalore | 119 | 9 | - | 2 | - | - | - | - |
| Kharibam, P., 2021 | Rishikesh, Uttarakhand | 1 | 1 | - | 1 | - | - | - | - |
| Mehta. V, 2019 | Rishikesh, Uttarakhand | 1 | 1 | - | 1 | 1 | - | - | - |
| Sachu A, 2018 | Alappuzha, Kerala | 193 | 33 | - | - | - | - | 1 | - |
| Raina S, 2018 | Himachal Pradesh | 11 | 3 | - | 1 | 1 | - | - | - |
| Diwan, A.G., 2014 | Pune, Maharashtra | 1 | 1 | - | - | 1 | - | - | - |
| Samanta, 2014. | Lucknow, Uttar Pradesh | 1 | 1 | Yes* | 1 | 1 | - | - | - |
| Chopdekar, 2014 | Mumbai | 2 | 2 | - | - | - | 7 | - | - |
| Singh R, 2014 | Lucknow, Uttar Pradesh | 1 | 1 | 6 | 1 | - | - | 1 | - |
| Damodar T, 2013 | Mangalore | 1 | 1 | 28 | 1 | 1 | 30 | 1 | - |
| Mahajan S.K, 2012 | Shimla, Himachal Pradesh | 1 | 1 | - | - | - | 7 | - | - |
| Kumar A, 2012 | Kochi, Kerala | 287 | 17 | - | - | - | 8.6 | 5 | 42 |
| Singh M P, 2012 | Chandigarh | 1 | 1 | - | - | - | - | 1 | - |
| Baliga, 2011 | Pune, Maharashtra | 2 | 2 | - | - | - | 14 | - | - |
| Gurjar, M., 2011 | Lucknow, Uttar Pradesh | 3 | 3 | 21.6 | 1 | 3 | 21.6 | - | - |
| Behera, B., 2010 | New Delhi | 1 | 1 | - | 1 | - | 31 | - | - |

[*Patient was admitted to the ICU, but the duration of stay was not documented in the included study]

**Table S10: Antibiotic administered in patients with leptospirosis co-infection**

| **Author/Year** | **Doxycycline** | **Ceftriaxone** | **Crystalline penicillin** | **Chloroquine** | **Azithromycin** | **Fluoroquinolones** | **Ofloxacin** |
| --- | --- | --- | --- | --- | --- | --- | --- |
| Shiddapur, G, 2024 | 1 | - | - | - | - | - | - |
| Nazeera NN, 2024 | 1 | 1 | - | - | - | - | - |
| Shiddapur G, 2024 | 1 | - | - | - | - | - | - |
| Aina, N, 2023 | 1 | 1 | - | - | 2 | 1 | 1 |
| Saboo K, 2023 | 1 | 1 | 1 | - | - | - | - |
| Gupta, N., 2022 | 16 | 17 | 11 | - | 5 | - | - |
| Dhanashree, B., 2021 | 9 | - | 5 | - | - | - | - |
| Kharibam, P., 2021 | 1 | - | - | - | - | - | - |
| Singh, A,2021 | 1 | - | - | - | 1 | - | - |
| Dev, N., 2019 | 1 | - | - | - | - | - | - |
| Mehta. V, 2019 | 1 | 1 | - | - | - | - | - |
| Negi, A., 2018 | - | 1 | - | - | - | - | - |
| Sachu, A., 2018 | - | - | 33 | - | - | - | - |
| Diwan, A.G., 2014 | 1 | 1 | - | - | - | - | - |
| Samanta, 2014. | 1 | 1 | - | - | - | - | - |
| Chopdekar, 2014 | - | - | 1 | - | - | - | - |
| Singh R, 2014 | - | 1 | - | - | - | - | - |
| Sharma S, 2014 | - | - | 8 | 8 | - | - | - |
| Damodar T, 2013 | 1 | 1 | - | - | - | - | - |
| Mahajan S.K, 2012 | 1 | 1 | - | - | - | - | - |
| Kumar, A, 2012 | 17 | - | 17 | - | - | - | - |
| Singh, MP, 2012 | - | 1 | - | - | - | - | - |
| Baliga, K.V., 2011 | 1 | 1 | - | 1 | - | - | - |
| Gurjar, M., 2011 | 1 | - | - | - | - | - | - |
| Behera, B., 2010 | - | 1 | - | - | - | - | - |
