# Supplementary material for: Leptospirosis and its co-infections in India: prevalence, geographic distribution, and clinical implications
Source: BMC Infect Dis. 2026 Jun 26;26:1653. doi: 10.1186/s12879-026-13642-7 (PMC13579849; doi:10.1186/s12879-026-13642-7)
Supplement: Supplementary file 2 [file 12879_2026_13642_MOESM2_ESM.docx]

**Supplementary File – 2**

**List of figures**

| Figure S1.a | Risk of bias assessment by JBI’s critical appraisal checklist for Studies Reporting Prevalence Data |
| --- | --- |
| Figure S1.b | Risk of bias assessment by JBI’s critical appraisal checklist for case reports |
| Figure S1.c | Risk of bias assessment by JBI’s critical appraisal checklist for Case series |

Checklist for Studies Reporting Prevalence Data

|  | Yes | No | Unclear | Not Applicable |
| --- | --- | --- | --- | --- |
| Was the sample frame appropriate to address the target population? | 15 | 0 | 0 | 0 |
| Was the response rate adequate, and if not, was the low response rate managed appropriately? | 9 | 3 | 3 | 0 |
| Was there appropriate statistical analysis? | 10 | 5 | 0 | 0 |
| Was the condition measured in a standard, reliable way for all participants? | 13 | 1 | 1 | 0 |
| Were valid methods used for the identification of the condition? | 15 | 0 | 0 | 0 |
| Was the data analysis conducted with sufficient coverage of the identified sample? | 15 | 0 | 0 | 0 |
| Were the study subjects and the setting described in detail? | 15 | 0 | 0 | 0 |
| Was the sample size adequate? | 13 | 1 | 1 | 0 |
| Were study participants sampled in an appropriate way? | 13 | 0 | 0 | 2 |

**Figure S1.a: Risk of bias assessment by JBI’s critical appraisal checklist for Studies Reporting Prevalence Data**

Checklist for case reports

|  | Yes | No | Unclear | Not Applicable |
| --- | --- | --- | --- | --- |
| Were patient’s demographic characteristics clearly described? | 20 | 0 | 0 | 0 |
| Was the patient’s history clearly described and presented as a timeline? | 20 | 0 | 0 | 0 |
| Was the current clinical condition of the patient on presentation clearly described? | 20 | 0 | 0 | 0 |
| Were diagnostic tests or assessment methods and the results clearly described? | 20 | 0 | 0 | 0 |
| Was the intervention(s) or treatment procedure(s) clearly described? | 18 | 0 | 2 | 0 |
| Was the post-intervention clinical condition clearly described? | 19 | 0 | 1 | 0 |
| Were adverse events (harms) or unanticipated events identified and described? | 4 | 16 | 0 | 0 |
| Does the case report provide takeaway lessons? | 17 | 2 | 1 | 0 |

**Figure S1.b: Risk of bias assessment by JBI’s critical appraisal checklist for case reports**

Checklist for Case Series

|  | Yes | No | Unclear | Not Applicable |
| --- | --- | --- | --- | --- |
| Was statistical analysis appropriate? | 2 | 2 | 0 | 0 |
| Was there clear reporting of the presenting site(s)/clinic(s) demographic information? | 1 | 2 | 1 | 0 |
| Were the outcomes or follow up results of cases clearly reported? | 3 | 1 | 0 | 0 |
| Was there clear reporting of clinical information of the participants? | 4 | 0 | 0 | 0 |
| Was there clear reporting of the demographics of the participants in the study? | 3 | 1 | 0 | 0 |
| Did the case series have complete inclusion of participants? | 3 | 0 | 1 | 0 |
| Did the case series have consecutive inclusion of participants? | 3 | 0 | 1 | 0 |
| Were valid methods used for identification of the condition for all participants included in the case series? | 4 | 0 | 0 | 0 |
| Was the condition measured in a standard, reliable way for all participants included in the case series? | 4 | 0 | 0 | 0 |
| Were there clear criteria for inclusion in the case series? | 3 | 0 | 1 | 0 |

**Figure S1.c: Risk of bias assessment by JBI’s critical appraisal checklist for Case series**
